# “I Wouldn’t Believe Her at First”—A Qualitative Study of Young People’s Sexual Consent Perceptions and Negotiation in Nairobi Informal Settlements

**DOI:** 10.1177/08862605231185301

**Published:** 2023-07-11

**Authors:** Phoene Mesa Oware, Katrine J. C. De Angeles, Wendy Ntinyari, Nickson Langat, Benjamin Mboya, Anna Mia Ekström, Anna E. Kågesten

**Affiliations:** 1 University of the Western Cape, Cape Town, South Africa; 2 Department of Global Public Health, Stockholm, Sweden; 3 Ujamaa-Africa, Nairobi, Kenya; 4 Department of Infectious Diseases, South Central Hospital, Stockholm, Sweden

**Keywords:** adolescent victims, sexual assault, prevention, sexual assault, sexual harassment, adolescents, sexual harassment

## Abstract

Forced or coerced sexual experiences have serious consequences for young people’s health and well-being. Healthy sexual consent communication can foster positive intimate relationships and help prevent unwanted sexual experiences. We aimed to explore how young people in Nairobi’s informal settlements construct, communicate, and negotiate sexual consent within heterosexual partnerships, given the limited insight into such experiences from resource-poor, global-south contexts. A qualitative study with young men and women aged 15 to 21 years was conducted among former participants of a school-based sexual violence prevention intervention in four informal settlements (slums) of Nairobi. Twenty-one individual in-depth interviews (*n* = 10 females, *n* = 11 males) and 10 focus group discussions (five with *n* = 6–11 males vs. females, respectively), that is, *n* = 89 in total were conducted. Data were analysed using thematic network analysis and interpreted using the Sexual script theory. Participants’ endorsement of incongruent sexual scripts shaped their perceptions and negotiations of sexual consent. Young men were committed to respecting sexual consent, but promoted male (sexual) dominance, and perceived women’s refusals as token resistance. Per traditional scripts of sexual chastity, young women were largely bound by their use of a “soft no” to give consent, so as to not display direct sexual interest. Actual non-assertive refusals thus risked being interpreted as consent. Young women’s “actual” refusals had to be more assertive (saying a “hard no”) and were described as having been influenced by skills learned during the school-based intervention. Findings highlight the need for sexual consent education to address internalized gendered norms about female token resistance, destigmatize female sexuality, reduce male dominance norms, and encourage young people’s respect for both assertive and non-assertive sexual consent communication.

## Introduction

Sexual violence remains a huge global public health problem, affecting nearly one in three girls and women—with rates being particularly high in sub-Sahara Africa (SSA) (World Health Organization [WHO], 2021), and further exacerbated by the COVID-19 pandemic lockdown measures (UN Women, 2021). Sexual coercion—the use of various strategies to force or pressure an individual to engage in sexual activities irrespective of their explicit or implicit refusal (Benbouriche & Parent, 2018) is a form of sexual violence that is a key concern in young people’s intimate relationships (Hird & Jackson, 2001). Coerced sexual experiences are linked to various negative health outcomes including increased risk of unplanned pregnancy, sexually transmitted infections, and poor psychological well-being (Nguyen et al., 2019; WHO, 2012).

Evidence from urban SSA contexts of Kampala (Uganda) and Johannesburg (South Africa) indicates a past-year incidence of sexual coercion among young women of 24% (15–24 years) and 27% (average age 21 years), respectively (Mokgatle & Menoe, 2014; Tusiime et al., 2015)—suggesting that at least one in four young women experience sexual activity without their consent in these contexts. Additionally, data from the nationally representative Violence Against Children Surveys conducted in nine low-income countries, of which eight are located in SSA, shows that during the 2007 to 2018 period, between 15% and 39% of females ages 13 to 24 years reported forced sexual initiation due to physical or psychological force (Howard et al., 2021). Similar estimates have been found for Kenya during the same period and in 2019, where 23% and 33% of 13 to 24 and 13 to 17-year old females respectively, reported forced sexual initiation (Howard et al., 2021, Republic of Kenya, 2019).

Sexual consent, which is the sober, conscious, and voluntary expression of willingness to engage in specific sexual behavior within a specific context (Willis & Jozkowski, 2019), is key in preventing sexual coercion and fostering positive sexual interactions (Brady et al., 2022; Muehlenhard et al., 2016). Current understandings of young people’s sexual consent communication are predominantly based on literature from Western contexts (Meyer et al., 2021), while evidence from low-income and lower-middle-income SSA contexts like Kenya (Meyer et al., 2021) is missing. In response to this gap, this study explores how young women and men (15–21 years) in Nairobi’s informal settlements, construct, communicate, and negotiate sexual consent within heterosexual intimate partnerships.

### Young People’s Perceptions and Negotiation of Sexual Consent

Previous studies indicate that young people perceive sexual consent as conscious mutual agreement or willingness to engage in sexual activity without force and coercion (Beare & Boonzaier, 2020; Bednarchik et al., 2022; Brady et al., 2018; Holmström et al., 2020; Jozkowski et al., 2014). Studies further suggest that young people recognize the importance of establishing sexual consent, and perceive the absence of consent as a form of sexual violence (Whittington, 2021; Wignall et al., 2020).

Evidence on whether and how young people negotiate sexual consent within heterosexual (steady) relationships paint a far more complicated picture. Previous studies suggest that sexual consent communication within relationships is a dynamic process, in which a wide array of signals including direct verbal (e.g., saying “no” or “stop”) and non-verbal cues (e.g., non-resistance to a partner’s sexual advances), indirect verbal (e.g., asking for a condom) and non-verbal cues (e.g., kissing, touching, and reciprocating a partner’s actions) are used (Beres, 2010, 2014; Hickman & Muehlenhard, 1999; Meyer et al., 2021). Overwhelmingly, young people rely on implicit verbal and non-verbal cues to communicate consent with their partners (Beres, 2010; De Meyer et al., 2022; Hickman & Muehlenhard, 1999; Jozkowski & Willis, 2020; Righi et al., 2021). Direct verbal sexual consent communication is often thought of as “awkward,” “taboo,” “impractical,” or as a “turn off” (Curtis & Burnett, 2017; Holmström et al., 2020; Wignall et al., 2020). Non-consent is equally communicated indirectly, via implicit cues such as pulling away and using indirect verbal statements such as stating that one does not have a condom, is on their period, is not ready, or has a boyfriend (Beres, 2010; Kitzinger & Frith, 1999; Righi et al., 2021).

Young people’s interpretation of sexual consent cues also appear to depend on context and situation (Beres, 2010; Wignall et al., 2020). For instance, in the context of a nightclub, willingness to transition to a private place can be interpreted as consent (Beres, 2010; Jozkowski & Willis, 2020). However, studies also suggest that gendered differences in men’s and women’s perception of consent cues can led to miscommunication. For instance, studies conducted among undergraduate students (Jozkowski et al.,2014) and among adult men and women (aged 19–72) (Newstrom et al., 2021) indicate that women (more than men) tend to use direct verbal strategies to indicate consent and non-consent with partners, even though men are more likely to interpret non-verbal cues. For the most part, however, young men and women are often attuned to each other’s consent cues within heterosexual partnerships, as a consequence of their socialization into gendered expectations and norms of sexual consent communication (Beres, 2010; Kitzinger & Frith, 1999).

### Theoretical Grounding

Our study is grounded in the sexual script theory developed by Simon and Gagnon (1986). They describe sexual scripts as norms that are acquired and internalized through socialization as a form of gendered guidelines (scripts) for men and women to engage in romantic and sexual interactions, including sexual consent communication (Simon and Gagnon, 1986; Wiederman, 2005). In most cultural settings, a traditional sexual script exists that promotes men as sexually assertive and dominant, and women as passive and subservient to men’s needs, leading to difficulties in communication about sexual consent (Zuo et al., 2018). Young women from SSA (Beare & Boonzaier, 2020; De Meyer et al., 2022; O’Sullivan et al., 2006) as well as from Western contexts (Burkett & Hamilton, 2012; Jozkowski et al., 2017) have described feeling obligated to consent to unwanted sexual relations.

Traditional gendered sexual scripts promoting women’s sexual restraint (Hird & Jackson, 2001; Jozkowski et al., 2014; Lewis et al., 2022; Wiederman, 2005) are also linked with norms of token resistance, that is, that women should refuse sex even if it is wanted, to protect their reputation and avoid social stigma (Jozkowski et al., 2017; Muehlenhard & Hollabaugh, 1988; Muehlenhard & Rodgers, 1998). Meanwhile, young men’s belief in token resistance (i.e., that a girl means “yes” even if she says “no”) has been linked with perpetration of sexual coercion, reflecting the complexity of seeking, negotiating, and respecting consent (Jozkowski et al., 2017; Lewis et al., 2022; Shafer et al., 2018). Traditional sexual scripts that uphold token resistance, male dominance, and female subservience are especially prevalent in many SSA settings including Kenya (Igundunasse & Odiase, 2021; Maticka-Tyndale et al., 2005; Wood et al., 2007)). In addition, young people’s sexuality in such contexts tends to be viewed as problematic, risky, and inappropriate, with sexuality education commonly promoting abstinence while overlooking relational aspects such as sexual consent communication (Sidze et al., 2017).

### Study Aim and Rationale

In this paper, we present findings from a qualitative study conducted with former participants in a school-based, behavioral intervention to prevent sexual assault in Nairobi urban informal settlements, Kenya. We aimed to understand how young women and men in this context construct, communicate and negotiate sexual consent with intimate heterosexual partners. We examine the ways that our participants drew on prevailing sexual scripts in their social context to inform their perceptions and negotiation of sexual consent.

## Methods

### Study Design, Setting, and Participants

This qualitative study was conducted among former participants in a behavior-based intervention to prevent sexual assault in Nairobi informal settlements. Implemented by the non-governmental organization Ujamaa-Africa (https://www.ujamaa-africa.org/), the intervention targets males and females in primary and secondary schools in high-risk contexts including urban informal settlements. The 6-week intervention comprises five parallel but complementary 2-hr interactive sessions for boys and girls, and a joint sixth session. The girls’ curriculum, IMPower, uses empowerment self-defence techniques to bolster confidence and self-esteem, critical reflection, and problem-solving skills including boundary setting, verbal assertiveness, negotiation, and self-defence, to enable girls to recognize and resist sexual harassment and sexual assault. The boys’ curriculum—Your Moment of Truth supports boys’ development of positive and non-violent masculinities by teaching skills in identifying emotions, bystander intervention, and understanding and seeking consent.

Previous evaluations using randomized controlled designs has found that the intervention can effectively reduce the incidence of sexual assault among girls (Baiocchi et al., 2017; Sarnquist et al., 2014; Sinclair et al., 2013), and improve boy’s gender attitudes as well as bystander intervention when witnessing gender-based violence (Keller et al., 2017). Findings from a qualitative evaluation conducted by our team to investigate the intervention’s mechanisms of change to prevent sexual assault further confirm this effect, as former participants reported feeling empowered to resist sexual assault using various strategies including negotiating sexual consent (Kågesten et al., 2021).

Data for the current study were drawn from semi-structured interviews conducted as part of the above-mentioned qualitative evaluation. Former intervention participants were recruited from five schools in different informal settlements in Nairobi (Kibera, Dandora, Viwandani, and Huruma). Participants were purposefully sampled based on their participation in a minimum of four sessions, at least 1 year before data collection. We conducted a total of 21 in-depth interviews (IDIs) (10 females, 11 males) and 10 focus group discussions (FGDs) with 6 to 10 participants in each group (five FGDs with a total of 46 males, and five FDGs with a total of 43 females) between January and June 2019 (Table 1).

**Table 1.** Number of Interviews, by Location, Method, and Participant Sex.

Location	Sex	Focus-Group Interviews	In-Depth Interviews
Kibera	Males	1 (8 participants)	2 participants
	Females	1 (8 participants)	1 participant
Dandora	Males	1 (11 participants)	4 participants
	Females	1 (10 participants)	5 participants
Mukuru-viwandani	Males	2 (8 participants)	4 participants
	Females	2 (8 participants)	4 participants
Huruma	Males	1 (11 participants)	1 participant
	Females	1 (9 participants)	—
**Total**	Males	5 (46 participants)	11 participants
	Females	5 (43 participants)	10 participants

### Data Collection

Interviews were conducted using structured interview guides in English, Kiswahili, and the local slang (sheng’) popularly used by young people. FGD participants were asked to reflect on traditional sexual scripts, based on the statement “when a girl says ‘no’ she means ‘yes’,” used to elicit further discussion on perceptions of gender norms related to masculinities and femininities, sexual violence, and sexual consent. Emerging themes were further explored through IDIs that focused on personal views and experiences of negotiating sexual consent with intimate partners. Interviews were audio-recorded, transcribed into the local language(s), and translated into English. Designated note-takers in FGDs took extensive notes during and after the interviews, using templates that prompted data collectors’ reflections on the interview context, participants’ non-verbal cues, emerging and unexpected themes. Notes and observations were discussed with the larger research team to identify themes for further exploration in IDIs.

To minimize power differentials and foster open discussions on gender and sexuality between study participants and data collectors, all interviews were conducted by trained, young (<30 years) data collectors who were of the same sex as the participants, from the study area and spoke the local language(s). All data collectors underwent a week-long training in qualitative methodology, research ethics, sampling, recruitment, interviewing skills, reflexivity, FGDs, and IDIs (practiced via interactive role-plays) conducted by researchers from Karolinska Institutet. The training was followed by field practice and piloting of interview guides with a smaller sample of *N* = 23 youth in the study setting under close supervision of more experienced members of the research team. Based on the results from the pilot, a 3-day refresher training was held just before data collection began.

### Ethical Considerations

Ethical approval was obtained from the National Commission for Science, Technology, and Innovation (NACOSTI) in Kenya (NACOSTI/P/18/73612/23379). Parental consent was waived to encourage participation from young people in discussing sexual behavior, which is a stigmatized topic in the research context (Abdallah et al., 2017; Bastien et al., 2011), and to avoid exacerbating participants’ risk of sexual violence in case their vulnerability was related to their home environment. We used various mechanisms to ensure the safety, confidentiality, privacy, and comfort of participants including written informed consent (assent for those under 18 years), witnessed by headteachers, and parental opt-out forms which allowed parents/guardians to object to their child’s participation in the study if under age 18. In case of distress following the disclosure of sexual assault, affected participants would be referred to support services for sexual assault survivors. All participants were provided with contact information for local child protection services and sexual and gender-based violence support services.

### Data Analysis

Data were analysed using Attride-Stirling’s (2001) thematic network analysis. An inductive approach was used to organize (code) the data in several steps. First, initial interview transcripts and field notes were coded by the lead author (PMO). Secondly, two researchers (PMO, AK) discussed, refined, and defined the codes, which were then applied to all remaining transcripts. For the current analysis, we focused on coded segments with content related to sexual consent communication and negotiation. Third, the main issues discussed within each code were identified and categorized into basic themes, which were further abstracted into organizing themes and global themes, forming a thematic network. The network was verified against coded texts to ensure the representation of identified themes in the data. During this process, gendered patterns and differences between narratives were also explored between male and female participants. All analyses were conducted using ATLAS.ti (version 9).

## Results

We identified two global themes with two corresponding organizing themes (i.e., sub-themes), respectively. The first global theme focuses on *young men’s perceptions of sexual consent*, including the organizing themes; (a) young men’s endorsement of the need to respect sexual consent, and; (b) young men’s hesitation to respect sexual consent. The second global theme focuses on *young women’s communication of sexual consent*, organized into; (a) perceptions that women communicate sexual consent indirectly, and; (b) non-verbal cues as qualifiers of non-consent (saying “no”). Below we present the findings in relation to each global theme and organizing theme, using pseudonyms in place of participants’ names to protect confidentiality.

### Young Men’s Perceptions of Sexual Consent

#### Young Men’s Endorsement of the Need to Respect Sexual Consent

Throughout the interviews, male participants consistently acknowledged the need to respect sexual consent. Reflecting on the statement “when a girl says ‘no’, she means ‘yes’,” most males emphasized the importance of respecting initial refusal of sexual consent without further speculation over its dual meaning. Jesse, a male FGD participant stated that: “. . .*if you ask, then she says ‘no’ you just have to understand*.” Job, another male IDI participant added: “*If I seduce a girl right now and she says ‘no’, I just let her go (. . .) now—I mean, there is no need to force her when she doesn’t want. Yeah*.”

Male participants’ narratives revealed their awareness and understanding of the value of respecting sexual consent, as well as their recognition of circumstances where consent cannot be given. As reflected in the below quotes, participants underscored that sexual consent is present when sex happens without force, or, through persuasion after a partner’s initial refusal.

I will have to understand her because I have learnt a lot and I spend time with girls, and I know them. If she says “no” and I insist and then she gives in that will still be rape, because she is not fully willing. (Martin, male IDI participant)

I feel like when I tell her and she refuses and then I [still] make her give in, I feel like I have forced her and have not respected her opinion. (Maxwell, male FGD participant)

The narrative by Chris below further reflected participants’ understanding that consent cannot be given by unconscious individuals.

For him [who has not been part of the intervention], you find that you have gone to town, to a club, you meet a girl, he brings the girl to his house, then they have sex. The girl is unconscious, she doesn’t understand, you see. I have found myself in such a situation (. . .) [but] I have already attended (. . .) training, I know. You see. So in my head, I am thinking, (. . ..) She has not agreed, you see, she cannot understand herself, this is not her home, you see. (. . .). This guy thought that I had had sex with this girl but I didn’t do it. (Chris, male IDI participant)

Participants like Chris linked his understanding of consent with the intervention, distinguishing himself from his peers who had not undergone the intervention and therefore lacked similar awareness about sexual consent. This was echoed by other participants, stating that:

I think it [ignoring consent] is a very bad thing, because when we learnt [as part of the intervention] that consent. . .it is there to help us to ask for permission. (Jesse, male FGD participant)

Overall, male participants’ narratives and perceptions of sexual consent demonstrated their exposure, awareness, and endorsement of an alternative sexual script to respect sexual consent.

#### Young Men’s Hesitation to Respect Sexual Consent

However, irrespective of the view that consent should be respected unconditionally, male participants also shared contradictory experiences and views. Despite acknowledging that consent should not be obtained through persuasion, participants narrated rarely respecting a female partner’s initial “no.” Often oblivious of their contradictions, they described respecting consent only after trying a range of tactics to either verify or reverse a partner’s initial refusal.

You know, because I am not used to that [a girl says “no”], I wouldn’t believe her at first. If she told me “I don’t want” I would tell her “You are just kidding me. You will eventually give in.” But if she doesn’t give in, I will respect that, I will tell her it is okay. Because we were taught by [the intervention] that if a girl says she’s not interested, tell her it is fine. Leave her alone. (Faraji, male IDI participant)

If she says “no,” I will ask again three times and if she says “no” again then I know that she means it (. . .) Just to confirm. (Maxwell, male FGD participant)

While male participants described their inclination to eventually respect consent, their narratives demonstrated the complex ways in which incongruent sexual scripts manifested in their consent communication practices.

Although not necessarily in intimate partnerships with their male peers, experiences shared by female participants affirmed narratives by their male counterparts, who described that sometimes refusals were met with “insisting and nagging” from a male partner.

. . .but you know for a guy if he seduces you and you say “no,” he will keep insisting, as most do not lose morale [do not give up], he will keep approaching you each and every time (. . .) every day (. . .) and you will end up accepting . . . like that. (Lilian, female FGD participant)

As for me, I can say, if that man approaches and hits on you and you tell him “NO!,” but he keeps insisting, you will find out that he just does not give up. So you end up telling him “yes” just to stop him from wasting your time. (Risper, female FGD participant)

Some male participants showed commitment to respect such a “no,” but were equally influenced by traditional scripts, that is, to only do so after ruling out their partner’s engagement in token resistance, which in turn underpinned experiences of sexual coercion. As illustrated, participants’ narratives about consenting to unwanted sexual interactions under pressure highlighted the influence of traditional sexual scripts and their potential to undermine new discourses on respecting consent.

### Young Women’s Communication of Sexual Consent

#### Young Women Communicate Sexual Consent Indirectly

Overall, participants narrated that young women use indirect cues to provide sexual consent, with both male and some female participants agreeing with the statement, “when a girl says ‘no’, she means ‘yes’.” Participants described (young) women’s refusal of (men’s) sexual advances as something normative, due to women being “shy,” that they “like pretending”; and that they lack the confidence to express their sexual desire and interest.

. . . (laughs) you know the girl is horny but is afraid to tell her boyfriend that she wants it [sex]. So when the boy suggests they have sex, the girl says “no, no” [speaks in a small voice] as she blushes, “yea.” (Julie, female IDI participant)

She can say yeah, “yes,” she wants something but she doesn’t have the confidence of saying “yes.” She may feel a certain way but it’s just that she’s afraid to say what she wants. (Job, IDI participant)

Some female participants however explained that their verbal refusals reflected their internal state and feelings and should therefore be unquestionably respected.

If it is “no,” I know the reason why I have said so. If it is “yes” I know why I have said “yes.” So I know if it is “no” it is “no” if it is yes it “yes”. . . Coz’ if I do not want (to do it), I don’t, if I want I want. (. . .) Yes, so don’t put me in something I do not want (to do). (Dionne, female IDI participant)

Rather than directly consenting to wanted sexual relations, participants described young women’s expressions of sexual interest as covert, implied via suggestive glances, “revealing” dressing, or regular and unplanned visits.

She cannot come [visit her partner] daily; she doesn’t come daily. When she comes, it is when she wants to have sex, but she will never come and tell you. You are the one to decipher. (Chris, male IDI participant)

if she is a girl who wants sex, you will see how she has dressed as in she has dressed on something tight and short then *inapandapanda* [meaning the dress moves upwards and she keeps on pulling it downwards] and she has put on sleeveless [top]. (Harriet, female FGD participant)

Through this quote, Harriet mirrored an internalized norm among study participants in this informal settlement that associates wearing “revealing” clothes with sexual consent, which can potentially aggravate the risk of sexual violence for women who dress in a certain way.

Women’s deviation from the traditional script (consenting to sex explicitly rather than not wanting it) was interpreted negatively. Frank, a male FGD participant for example stated that: “*in a relationship, issues about sex, the boy is supposed to initiate. Actually, it’s because boys are the heads and controllers*.” Frank’s view illustrates participants’ sentiment that young women’s direct communication of consent through actively initiating sexual activity transgressed acceptable gender norms about sexual interactions. This was further emphasized by Robert, a male IDI participant who described girls’ initiation of sex as suspicious, indicating sexual risk behavior, which would force him to take extra precaution: “*on that now. . . in that situation, I must use a condom*.”

Participants also described societal forces underpinning women’s inclination to invariably “say no,” including when sex is wanted. Female and male participants alike described social consequences for young women’s engagement in intimate relationships, including disapproval from parents or guardians who view such conduct as “going astray,” or reputational damage for consenting to sexual relations too quickly, including being labeled “too easy,” “cheap,” “loose,” or “a whore.”

She might say—she might say—if she directly says “yes,” somebody might think she is an easy girl and leave. (Job, male IDI participant)

Okay, you can find a situation where a girl says “no” yet she means “yes,” for example, if [I] have gone to a place and a man who is a friend to [you] hits on me for the first time, I will say “no” (. . .) I am not cheap but back in the mind have accepted am saying “yes! Yes! Yes!”(. . .) Because I do not want [you] to take me for less, to see me as cheap. (Lucy, female FGD participant)

Further, some female participants indicated that women’s use of contradictory consent cues is a manifestation of an internal dilemma that can stem from young women’s negotiation of the perceived sexual risks (e.g., pregnancy) and rewards (e.g, fulfillment of sexual desires). To this end, Lorraine, a FGD participant stated. “*Anyway, there are some people, they say ‘no’ and yet they mean ‘yes’ because they have mixed feelings, yeah*.” Nuru elaborated:

Saying “no” meaning “yes,”. . .so now it reaches a point as in you are with your boyfriend, right, you mean “no” and at the same time you mean “yes”. So now that’s how you find one being impregnated by her boyfriend because in the situation they are in, she is saying “no” but has feelings for him so that “no” is meant to avoid pregnancy while the “yes” is for the feelings. (Nuru, female IDI participant)

The above quote illustrates how participants ascribed to the traditional sexual script that girls say “no” to mean “yes”; characterizing token resistance as normative—a natural flow of sexual interaction—and as a strategy to cope with perceived consequences of sexual engagement such as contempt from both peers and parents, and as a manifestation of young women’s negotiation of perceived risks and rewards of having sex.

#### Non-Verbal Cues as Qualifiers of Non-Consent (Saying “No”)

Participants narrated that paying attention to a partner’s body language, and (re)actions irrespective of their verbal non-consent was crucial. Male participants, despite their view that sexual consent refusals should be consistently and unconditionally respected, elaborated: “*Yeah. You look at her reaction when she’s saying ‘no’, you look at her reaction*” (Job, a male IDI participant). Gilbert, another male FGD participant stated “*Knowing if she has said ‘yes’, observing her behavior. . .*”.

According to both male and female participants, to be perceived as a credible non-consent, a female partner had to say “no!” assertively, in a “loud and firm” voice, while portraying seriousness such as by “frowning,” “maintaining eye contact,” “standing up to leave,” or “changing one’s mood.”

Let’s say, there was a time I asked my girlfriend, and. . . she said “no!” and it was with a very harsh tone. I could feel that this is serious, let me just let go. Yeah. (Job, male IDI participant)

Participant’s views about credible non-consent were further linked to the intervention, which they described as helping women to become more assertive in their refusals of unwanted sexual activities or intimate relations.

For a girl who has not passed through these sessions [the intervention] (. . .) she is not firm with her decision. At first, she is afraid, I mean fifty-fifty. (Linda, female FGD participant)

It depends on one’s confidence maybe one has gone through classes [the intervention] and that’s how she has slowly gained the confidence to say “No,” such a person just looks at you and says “No” and “No.” (Martin, male IDI participant)

While female participants described how the intervention helped them to become (more) assertive to stop sexual assault, for example by using their voices and bodies to communicate a “hard no”; these skills were less straightforward within intimate relationships. Female participants described that saying “no” can indicate both willingness and unwillingness to engage in an intimate relationship or sexual activities, and that non-verbal cues are central to unpacking its underlying meaning. Saying no “politely,” “in a low tone,” “while fidgeting,” or “smiling” were seen as sexual consent greenlights, clearly illustrated in one participant’s remark below.

Because there is a “no” that shows that one is serious. . . “NO!” and there are those that say “no” (. . .) “no” [low tone], so, the way I see it, there are two “no’s.” (Zawadi, female IDI participant)

To consent to wanted sexual and intimate relations, therefore, female participants invoked traditional sexual scripts that constrain women’s overt expression of sexual interest, that is, by saying a “soft” *no* rather than an explicit *yes*. Female participants also narrated that failing to use non-verbal cues, such as indicating seriousness, could result in being misunderstood as Julie, a female IDI participant lamented.

For me, if I say “no,” and I have never known how to—let us say, being serious, then the boy will just say “you want it.”

Participants’ perceptions and negotiations of sexual consent, therefore, drew from both traditional and alternative sexual scripts. Consenting to sexual relations was consistent with traditional sexual scripts—saying “no” less assertively to mean “yes”—while refusing unwanted sexual relations was consistent with alternative scripts, to say “no” assertively.

## Discussion

We set out to explore how young men and women (15–21 years), who participated in a school-based behavioral intervention to prevent sexual assault in Nairobi informal settlements, construct, communicate, and negotiate sexual consent with intimate partners. We found a complex ambivalence among participants, who clearly underscored the importance of seeking and accepting consent, while at the same time endorsed traditional sexual scripts of female token resistance. Our findings revealed that participants’ endorsement of incongruent sexual scripts shaped their perceptions and negotiations of sexual consent. Young men narrated the importance of respecting sexual consent, but as per traditional gendered scripts, also viewed women’s refusals as token resistance and an invitation for further persuasion. Young women’s “actual” refusals of unwanted sexual relations were described to be direct and assertive (saying a “hard no”), and were linked with the intervention which is designed to prevent sexual assault. However, within intimate relationships, young women mainly used non-verbal signals (a “soft no”) to consent to wanted sexual relations, so as to not display a direct sexual interest.

Overall, these findings confirm the limited literature on sexual consent communication among young people in non-western contexts, which highlights consent as a *process* that can occur directly or indirectly through both verbal and non-verbal signals (Meyer et al., 2021). Similar to recent qualitative research conducted with 15 to 24-years-olds in Uganda and Ecuador, young people in our study mainly used indirect signals to communicate consent in wanted sexual relations (saying “no” to mean “yes”), whereas direct signals were used to indicate non-consent (saying “no” to mean “no”) (De Meyer et al., 2022). This dynamic is also consistent with sexual scripts that have been reported in young people’s dating relationships in various SSA contexts, which do not allow young women’s direct consent to sex (Igundunasse & Odiase, 2021; Maticka-Tyndale et al., 2005). The fine line between these strategies is complex to decode and can easily be misinterpreted, as was the case in Uganda where lack of verbal boundary setting meant that sexual consent was “mostly assumed” (De Meyer et al., 2022). Given that sex is seen as obligatory on the part of women and an entitlement for men in many SSA context—which in turn fuels coerced sexual experiences (Maticka-Tyndale et al., 2005; Wood et al., 2007) —the fact that many young women in our study were able to apply verbal strategies as a result of skills learnt through the intervention—and that young men recognized the importance of seeking consent—is promising.

However, what becomes clear is that direct verbal consent did not always resonate with our study participants’ realities in light of prevailing sexual scripts, especially within intimate (steady) relationships. This is in line with findings from other studies that highlight how young people’s reasoning around sexual consent as direct and verbal is detached from their real-life enactment of consent as being more consistent with traditional scripts (Brady et al., 2018; Holmström et al., 2020; Righi et al., 2021; Wignall et al., 2020).

Our findings carry important implications for understanding young people’s sexual consent communication and guiding interventions to improve such practices in low-income contexts of Kenya and other SSA settings. Firstly, our findings highlight the need for interventions to address and challenge, at a social and structural level, the prevailing stereotypical gendered sexual scripts that are supportive of token resistance and sexual coercion. While participants endorsed the need to respect consent, we found that traditional gendered sexual scripts remain a powerful influence on sexual consent communication—affirming the link between young people’s internalization of myths about gender and sexuality and their endorsement of sexual coercion (Fernández-Fuertes et al., 2020; Hird & Jackson, 2001; Kilimnik & Humphreys, 2018; Shafer et al., 2018; Trottier et al., 2021; Zuo et al., 2018). Second, the voices of former intervention participants in our study highlight the significant challenge of transforming gender norms as a complex process, requiring multi-level efforts and actors (e.g., community, family, school) throughout childhood and adolescence (Levy et al., 2020). In this regard, we echo suggestions by other scholars for sexual violence prevention intervention strategies to move beyond the individual and target different demographics and social spaces where young people are socialized (De Meyer et al., 2021).

Third, our findings draw attention to a key gap in sexual consent communication: the need to adopt a sex-positive approach that destigmatizes girls’ and young women’s sexuality and engagement in consensual sexual experiences (De Meyer et al., 2022; Jackson & Cram, 2003; Thompson et al., 2020) and correspondingly, challenge norms of male sexual dominance given young men’s disapproval of sexually assertive women in this study. While interventions which enhance girls’ assertive refusal and resistance of unwanted sexual experiences can be valuable in preventing sexual violence (Baiocchi et al., 2017; Decker et al., 2018; Edwards et al., 2021; Kågesten et al., 2021), it is clear from our findings that scripts of female sexual token resistance prevail within intimate relationships. This was evident in female participants’ descriptions of refusing wanted intimate relations, which as this and previous research (Muehlenhard et al., 2016) have highlighted, perpetuates a willingness among young men to violate consent boundaries despite their understanding of respecting the very same. Sex-positive approaches that destigmatize young women’s sexuality can address barriers to young women’s direct consent to wanted sexual activities (Thompson et al., 2020) and subvert norms that underpin notions of miscommunication underpinning sexual violence (Motley, 2008).

Relatedly, these findings highlight a key limitation of the “no means no” discourse in sexual violence prevention; much like traditional sexual scripts (Wiederman, 2005); it positions women as gatekeepers (Gilbert, 2018; Mitchell et al., 1996), leaving them without a framework to consent to *wanted* sexual relations. Our findings, therefore, confirm the need to dismantle norms that undergird women’s compulsion to engage in token resistance (Pugh & Becker, 2018) and to further tackle female sexual stigmatization by fostering environments that allow young women to not only say “no” when they do not want something—but “*yes*” to *wanted* sexual interactions in line with their wishes and desires. To this end, a key consideration is the need to enhance young women’s access to sexual and reproductive health services, for example, contraceptives. This is in view of data that suggests moderate (59%) modern contraceptive use among sexually active unmarried women (15–49) in Kenya (Kenya Demographic and Health Survey [KDHS], 2022) and our finding which reveals that some young women’s use of contradictory consent cues is due to their internal conflict between fulfilling their sexual desire and concern over the consequences of sexual involvement like pregnancy.

Finally, our findings establish a need to re-examine emphasis on direct and assertive sexual consent communication. Lewis et al., (2022) note that “soft” refusals risk being dismissed in contexts where non-consent is only defined as aggressive resistance. Indeed, studies have found that young men rationalize sexual coercion after a woman’s refusal, citing women not being assertive enough (Burkett & Hamilton, 2012; Jozkowski et al., 2017). Reinforcing a standard where only assertive refusals are deemed legitimate is also problematic as socio-cultural norms can restrict women’s capacity to convey assertive refusals (Kitzinger & Frith, 1999). This was evident in one participant’s concern that her “innate” inability to communicate refusals assertively meant that her “no” would be misconstrued as willingness. It is important to recognize that strategies for communicating consent refusals often derive from cultural contexts and that consent cues, including the most subtle ones, are typically unambiguous and mutually understood by communicating parties (Beres, 2010, Hansen et al., 2010). Therefore, sexual consent education interventions should not only focus on building assertive consent communication skills but also on promoting awareness, recognition, and willingness to respect consent cues within the context of each individual’s experiences and culture (Harris, 2018).

### Limitations

Our findings and their implications are limited to the specific study context and cannot be generalized to all young people in Nairobi settlements, Kenya, or SSA more broadly. Further, the views and discussions by former participants of a sexual violence intervention around consent may differ from those without similar experiences. However, experiences of the participants in the current study highlight the complexity of consent communication, allowing findings to be (more) useful to guide the design, implementation, and qualitative evaluation of similar interventions in other contexts. Also, while our study provides key insights into consent communication practices among young men and women in the study context, it did not explore boys’ and young men’s experiences of giving sexual consent, their experiences of sexual coercion, or sexual consent communication practices for young men and women within non-heterosexual interactions. These are important venues for future research and programming, along with exploring the influence of socio-economic vulnerability and safety risks present in urban informal settlements on young people’s sexual consent communication practices.

## Conclusions

Finding from this study in Nairobi informal settlements highlight young people’s sexual consent communication as a complex and contradictory process involving direct and indirect verbal and non-verbal cues, grounded in incongruent sexual scripts. While young men were committed to respecting consent, they also assumed female token resistance; and while young women shared strategies to say a “hard no” where sex was clearly unwanted, they were also bound by their use of a “soft no” to mean *yes* within intimate relationships to avoid the stigma associated with female sexual desire. Our findings emphasize the need for sex-positive approaches that destigmatize young women’s sexuality, challenge stereotypical gender norms of expectations of male sexual dominance and debunk myths and practices of token resistance. It is also clear that behavior-based interventions aiming to improve young people’s ability to set and negotiate sexual boundaries need to be combined with structural and multi-level approaches in order to fully address the pervasive influence of social norms. Finally, we underscore the importance of encouraging young people to seek and respect consent under all circumstances including sexual relationships that are wanted, irrespective of whether consent is communicated assertively or non-assertively.
